# Ultra-Processed Foods and Metabolic Dysfunction: A Narrative Review of Dietary Processing, Behavioral Drivers and Chronic Disease Risk

**DOI:** 10.3390/metabo15120784

**Published:** 2025-12-05

**Authors:** Tyler J. Godsey, Travis Eden, Sam R. Emerson

**Affiliations:** Department of Nutritional Sciences, Oklahoma State University, Stillwater, OK 74078, USA; travis.eden@okstate.edu (T.E.); sam.emerson@okstate.edu (S.R.E.)

**Keywords:** ultra-processed foods, dietary processing, obesity, metabolic health, chronic disease, dietary quality, energy intake, NOVA classification

## Abstract

**Background/Objectives:** Ultra-processed foods (UPFs) have become a dominant component of the modern diet, paralleling the rise in obesity and chronic disease prevalence worldwide. This narrative review aims to synthesize evidence on how dietary processing and UPF consumption interacts with dietary quality, energy balance, and biological pathways to influence metabolic health. **Methods:** We performed a targeted literature search of peer-reviewed articles and authoritative reports examining UPF definition (via the NOVA classification), global consumption patterns, behavioral drivers of overconsumption, nutrient composition, and mechanistic links to metabolic dysfunction. Emphasis was placed on recent human and animal research relating UPFs to obesity, cardiometabolic outcomes, inflammation and gut microbiome alterations. **Results:** High UPF intake is consistently associated with reduced diet quality (higher saturated fat, sugar, sodium; lower fiber and micronutrients), increased energy density, faster eating rates and activation of reward pathways. These factors facilitate excessive energy intake and adiposity, promoting metabolic dysregulation, chronic low-grade inflammation, hormonal disturbances and gut microbiome shifts. While cross-sectional and cohort evidence is extensive, causal intervention trials and mechanistic human work remain limited. **Conclusions:** The accumulated evidence suggests that UPFs may influence chronic disease risk through their unbalanced nutrient profiles and through additional effects introduced by industrial processing. To translate these insights into public health strategies, future work should prioritize real-world intervention studies to reduce UPF consumption and examine resulting effects on energy balance, inflammation and gut health.

## 1. Introduction to Ultra-Processed Foods and Negative Health Consequences

Over half of the adults in the United States have at least one chronic disease, and roughly a quarter of adults have two or more [1]. These conditions impact patients for extended periods of their lives, require ongoing medical intervention, and include diseases like cardiovascular disease, diabetes, hypertension, obesity, and cancer. Heart disease, cancer, and diabetes are the leading causes of disability and death in the United States, and they are primary contributors to the $4.5 trillion annual health care cost in the US [2]. Although the etiology of chronic disease can be multifaceted, there is significant interest in modifiable risk factors such as diet, exercise, and lifestyle. A novel dietary approach includes the reduction in ultra-processed food (UPF) consumption, which has drastically increased in recent decades [3,4,5]. UPFs are industrially manufactured, ready-to-eat products that are highly energy dense, typically high in added sugars, saturated fat, and sodium, and low in protein and fiber [6]. Thus, it is hypothesized that UPF may be a major driver of the increased prevalence of chronic health conditions, including obesity, and modification of UPF consumption may impact metabolic health and minimize chronic health conditions.

## 2. Ultra-Processed Foods

### 2.1. Development of Ultra-Processed Foods

Modern food processing has been vital to human life by promoting food safety, ensuring food security, reducing food waste, and meeting nutritional needs, it also has its share of downsides [7]. To fully understand UPFs and the impact they have on our food system and population health, it is important to examine the history and development of these foods. Our current food system has undergone an extreme and accelerated evolution over the past ~70 years, likely contributing to the increased consumption of UPFs in the United States that has been observed in recent years [4]. This production and consumption of these UPFs can be traced back to the 18th century when economist Thomas Malthus predicted that the population growth would be too great for the agricultural capabilities of the time [8]. This concept, known as the Malthusian Trap, lit the first sparks for widespread concerns of famine and resource shortages [9]. While impactful for its time, this Malthus theory is limited by its assumption of static agricultural productivity, overemphasis on biological determinism, misconceptions on famine drivers (excluding food shortages), and fails to take into account human adaptability and innovation [10]. Several decades later, Nobel Prize-winning biologist Paul Ehrlich expressed opinions starting in the 1960s that the US would soon fall victim to these famines as soon as the 1980s [11]. However, to avoid the Malthusian Trap, in the 1900s new agricultural and industrial advancements in manufacturing, food packaging, and preservation contributed to an increase in processed foods, as well as an increased focus on productivity and specialization of farms [12,13,14,15]. This increased of farm specialization was fueled by US government incentives for increased commodity crops production and cheap ingredient production [14,16]. The abundance of these cheap ingredients paved the road for processed food companies to make more profitable products at a mass scale that were shelf-stable and hyperpalatable [16]. UPFs have become the culmination of these various advancements with the key characteristics of (1) convenience due to their ready-to-consume nature and shelf stability, (2) attractiveness due to the hyperpalatable taste and eye-catching packaging, and (3) highly profitable due to the low-cost ingredients used [17]. These favorable attributes, both to consumers and sellers, would be a major factor in explaining that the average US adult’s diet has a majority of daily calories (~57%) from UPFs, a number that has increased throughout the years [4].

### 2.2. Classification of Ultra-Processed Foods

Although there has been increased interest in UPFs in the literature and news, there is not a universally accepted and inclusive definition for these foods. This in turn has led to a mixed perception of what UPFs are, which generates more confusion, even among professionals [18]. To better navigate and categorize the extensive food market, Monteiro et al. introduced the NOVA system of classifying foods based on their level of processing [19]. The NOVA system emphasizes food processing under the justification that conventional methods (e.g., food origin or nutrient content) of classification no longer work well due to the sole focus on a food’s animal or plant origins, leading to a wide spectrum of foods within each category [17]. Another justification of this system is the increasing evidence that there is a relationship between food processing and various health outcomes. The 2020–2025 Dietary Guidelines for Americans states that the reduction in processed meats, saturated and trans fats, added sugars, sodium, and refined grains in the diet are ways of implementing healthy dietary patterns to avoid negative health outcomes, with the recommendation that these foods only contribute to 15% or less of one’s total daily caloric intake [20]. These recommendations do highlight aspects of foods that align with the NOVA classification system, though the document does not discuss the differences in processing that exist in current foods and the potential associated health consequences. Other justifications for a focus on food processing are motivated by the change in food systems/supplies on the global level, the financial benefit to international corporations to sell these products, and the level of manufacturing and promotion from processed food companies [17].

The NOVA system consists of four categories: unprocessed/minimally processed foods (Group 1), processed culinary ingredients (Group 2), processed foods (Group 3), and UPFs (Group 4) [21]. UPFs include sugar-sweetened beverages, pre-prepared meals, reconstituted meat and cheese products, and packaged ready-to-eat snacks. UPFs are industrial formulations of food-derived substances and additives with little resemblance or content of raw or unprocessed foods [17]. The NOVA definition of UPFs also includes the manufacturing steps that create the food products, including extraction, hydrogenation, preservation, stabilization, hydrolyzation, and emulsification [17].

Critiques of the NOVA system include the focus on food processing over formulation [22], the lack of evidence for UPF reduction being more suitable than other established dietary guidelines [23], and discrepancies among individuals and their perceptions of which foods belong to certain categories [24]. It would certainly be a difficult task for the food industry to reformulate or eliminate products based on the NOVA system’s definition of UPFs given the abundance of products that fall within the category. The idea that Group 4 foods need further differentiation based on different biological mechanisms and associated health outcomes to guide reformulation may be a possible solution for future policies and solutions [25]. Additionally, critiques of the NOVA system expand to studies that rely on the classification system for dietary intervention methods. A critique of the well-known Hall et al. clinical trial includes the drastic variability between the ultra-processed and unprocessed diets in terms of palatability, presentation and the source and form of food [26]. This critique has also been expanded to a similar study by Hamano et al. where the increased consumption could be explained by more palatable options for the UPF group compared to the unprocessed group, highlighting the need for investigators to carefully consider how they implement the NOVA system in their protocol [26]. Despite these critiques, the NOVA system has been validated by consistent classification between individuals and the utilization of the system in dietary intake literature [5,27]. Additionally, the NOVA system also avoids the well-established model of nutritionism in the field of nutritional sciences, which sets the primary focus on nutrient composition of foods for health outcomes [28,29]. Currently, the NOVA classification acts as a useful tool in the area of nutrition and public health to provide a well-structured definition, and it is an excellent way to assess the average UPF consumption in a population and the health consequences of high consumption [5,30].

### 2.3. Dietary Quality of Ultra-Processed Foods

The increased prevalence of UPFs in the average US adult diet has raised concerns and prompted investigations in the nutritional quality of diets in the United States [31,32]. Liu et al. reported that higher UPF consumption is associated with lower scores for the American Heart Association (AHA) diet score and Healthy Eating Index (HEI)-2015 score [33]. The industrial formulation of UPFs results in products that are typically high in added sugars, saturated fats, sodium, and artificial additives, while being low in fiber, protein, and other key nutrients [6]. Steele et al. observed that as UPF contribution to total calories increased, consumption of carbohydrates, saturated fat, and added sugar increased [31]. Further, they also saw that the increased UPF contribution decreased dietary fiber, protein, vitamin, and mineral consumption [31]. Meanwhile, individuals with a higher consumption of minimally processed foods see the opposite in their dietary quality, mainly through higher protein, fiber, and potassium intakes, highlighting the importance of lower processed food intake [34,35,36]. Diet patterns that focus less on UPFs, like the Mediterranean and Dietary Approaches to Stop Hypertension (DASH) diets, have shown health benefits to chronic disease prevention by lowering risk of cardiovascular events, type 2 diabetes, and cancer [37]. The effect that UPFs have on dietary quality appears clear and is a likely driving factor in the negative health consequences that are also seen with elevated UPF consumption.

The formulation of UPFs results in a food product that is far derived from the unprocessed or minimally processed foods that they are displacing [6]. This displacement increases the likelihood that an individual does not meet the recommended amounts of fruits, vegetables, whole grains, dietary fiber, as described by the Dietary Guidelines for Americans [20,33]. Globally and in the United States, UPF consumption is inversely associated with dietary fiber intake [31,32]. In the United States, more than 90% of adults do not meet the dietary fiber recommendations, mirroring the underconsumption of fruits, vegetables, and whole grains by about 85% of US adults [20]. Adequate consumption of dietary fiber contributes to many health benefits including improved insulin sensitivity, gut microbiota diversity, reduced chronic inflammation, and favorable gut health [38]. UPFs often contain a combination of ingredients and additives (e.g., emulsifiers, artificial sweeteners, colorants, stabilizers) that are not typically found in whole foods, which can also disrupt the gut microbiome [39]. Further, the additives may disrupt evolved nutrient-flavor associations [40]. This may be seen when consuming artificial flavors that provide sweetness in the absence of sugar or umami without protein being present [40]. This disruption in the flavor-nutrient relationship may dysregulate food intake and lead to weight gain [40]. In 1958, the US Food and Drug Administration (FDA) began to regulate food additives [40]. The list of additives started with 800 chemicals, but with the FDA allowing manufacturers to bypass premarket approval for substances that were considered generally recognized as safe (GRAS), there have been around 10,000 additives approved over the last 60 years [40]. Several research groups and public health organizations have argued that evaluating additives individually may underestimate risk, given that consumers are chronically exposed to complex mixtures of additives across multiple UPFs [41]. Groups such as the European Food Safety Authority (EFSA) [42], the Food Packaging Forum [43], and researchers identifying the “cocktail effect” of food chemicals [41] have argued for risk assessments that integrate mixture toxicity models rather than single-compound evaluations. Critics of the current system note that the GRAS pathway offers limited oversight of cumulative exposures, despite the fact that UPF consumption patterns lead to repeated daily intake of dozens of additives simultaneously [44]. Together, these concerns suggest that additives may play a broader role in UPF-associated metabolic risk than can be captured by evaluating each compound in isolation.

Although not lacking protein, UPF-rich diets have a diluted proportion of their calories contributed by protein, while simultaneously having more calories. This results in individuals who consume high amounts of UPF to over consume calories when aiming to meet protein intake recommendations [45]. The category of UPFs includes reconstituted meat products such as sausage, hot dogs, chicken and fish nuggets, and other “meat slurries”, which include meat scraps, trimmings, and other cheap cuts homogenized with various emulsifiers and stabilizers [46]. Much like dietary fiber, protein intake (proportion of total kcal) has been shown to decrease as one consumes more UPFs in the US and globally [31,45]. Additional formulated forms of protein include modified sources such as hydrolyzed proteins, soy isolate, gluten, casein, and whey protein [6]. Often, protein formulations lack protein quality due to the absence of essential amino acids or impaired digestibility and absorption [47]. Meanwhile, high-quality proteins can be found in unprocessed animal and plant sources such as eggs, salmon, lean meats, soybeans, and milk [47]. In support of consumption of high-quality proteins, minimally and unprocessed diets are associated with greater animal protein intake and plant protein diversity, and processed foods are associated with only greater plant protein intake [34]. Adequate protein consumption is crucial for all individuals for body growth and development while improving insulin sensitivity and combatting sarcopenia in older adults [48]. To further highlight the effect UPF consumption has on nutrient underconsumption, greater UPF intake is correlated with the decreased consumption of vitamins A, C, D, E, B3, and B12, as well as potassium, zinc, and magnesium [32]. The formulation of UPFs from unprocessed and minimally processed foods results in a lack of beneficial nutrients that are hallmarks of healthy diets, including dietary fiber, protein, vitamins, and minerals.

A major concern with the rise in UPF consumption is the increased intake of saturated fat, added sugar, and sodium [16]. The unique composition of UPFs that makes them more palatable includes their ratios of salt to fat, sugar to fat, and carbohydrate to salt, influencing the desire to consume these foods [7]. According to the Dietary Guidelines for Americans, only 23% of adults consume a healthy amount of saturated fat in line with the limit of 10% of daily calories [20]. Dietary patterns that reduce saturated fats or replace them with unsaturated fatty acids are associated with favorable cardiovascular health outcomes [49]. Main sources of saturated fat in the US include red meat, sandwiches, desserts, sweet snacks, and dairy-based mixed dishes, all of which are or can be UPF [20]. The rise in saturated fat consumption may in part be due to the increased use of crude palm oil and palm kernel oil in the manufacturing process of several UPFs including baked goods, cheese products, frozen meals, dressings, and more [50,51]. Added sugars are also overconsumed on average in the US at roughly 13% of total calories, exceeding the recommended 10% limit [20]. Increased added sugar consumption is associated with a myriad of health consequences including obesity, diabetes, cardiovascular disease, and cognitive decline, emphasizing the importance of moderate consumption [52]. The majority of the added sugar sources in the US are also classified as UPFs including sugar-sweetened beverages, desserts/sweet snacks, candy, coffee and tea [20]. Sodium is another essential nutrient that has been consumed in excess by US adults with an average consumption of 3393 mg, heavily exceeding the recommended upper limit of 2300 mg [20]. High sodium intake has been widely associated with increases in blood pressure, organ damage, and cardiac events [53]. Most sodium consumed in the US does not come from salt endogenous to foods, or even salt added at the table, but rather from the added salts in processed food products [20]. The main roles of sodium in food processing are flavor enhancement and preservation [54]. In the culinary setting, salt is a useful additive to appeal to the sensory aspect of food consumption by improving taste, and this pleasantness can be strengthened with further increases in salt [54]. Further, salt is a valued preservative due to its ability to prevent microbial growth by reducing water activity in foods, as well as its crucial role in food fermentation [54]. Overall, the goal and end result of these altered nutrient levels found in UPFs are food products that are hyperpalatable [55]. Highly palatable foods can offset appetite regulation by up-regulating hunger signals, blunting satiety signals, and activating the reward system, all of which drive consumption for pleasure instead of energy requirements [56]. The negative health associations of elevated levels of saturated fat, added sugar and sodium, along with the potential for palate-driven overconsumption, strengthen the concerns of increased UPF consumption and how it impacts health.

### 2.4. Ultra-Processed Foods and Energy Consumption

An additional characteristic of concern in UPFs parallel to that of the altered nutritional quality is the energy-dense nature of the food products [6]. Energy-dense foods have more calories per weight of food, and this is typically due to the abundance of fats and sugars which provide 9 kcal/g and 4 kcal/g, respectively, as well as the general lack of water in processed foods [57]. Over the past few decades, the prevalence of energy-dense foods (classified as >2 kcal/g) has increased from ~37% in the late 1980s to ~47% in 2018 [58]. In the US, recommended diets and guidelines emphasize the consumption of foods that are nutrient-dense and low-energy-dense [59]. These foods are able to have a reduced energy density while meeting nutrient needs due to high amounts of water (e.g., fruits and vegetables), fiber (e.g., whole grains and legumes), or protein (e.g., lean meat, poultry, and fish) in the food [60]. These characteristics of low-energy-dense, high-nutrient-dense foods can be beneficial to weight management by contributing less to total caloric intake and increasing satiety [61,62,63]. Additionally, foods that would be defined as both nutrient- and energy-dense (e.g., avocados, nuts, and cheeses) are beneficial for providing micronutrients, improving diet quality, and minimizing risk of chronic disease [64,65,66]. The water content in food also heavily influences its energy density, as it contributes to food weight and not caloric content [57]. UPFs typically have a lower water content compared to minimally processed foods, regardless of nutrient content [67]. The intentional dehydration process that causes low water content is favorable for producing desired food textures, palatable flavors, and extending the shelf life of products, while also increasing the energy density [67]. Further evidence of the relationship between UPFs and energy density shows that over two-thirds of energy-dense foods also fall under the definition of UPF, and this overlap percentage increased from 67% to 77% in a 30-year span [58]. In addition to heightened prevalence, the cost of high energy-dense foods (e.g., per 1000 kcal and per serving) are cheaper than low energy-dense foods [68,69]. Energy density is a significant characteristic of UPFs that reflects the altered nutrient status of the foods and likely contributes to overconsumption.

Energy consumption has also been established as being influenced by the rate of eating and the sensory characteristics of food [70]. Much like high energy density, faster eating rates are associated with a higher ad libitum energy intake compared to eating at slower rates [71]. Further, Hall et al. showed that when individuals are given a meal that comprises UPFs, they will consume it at a faster rate (g/min and kcal/min) compared to when they are given an unprocessed diet, resulting in greater total caloric intakes [72]. Food texture can be a major driver of eating rate, as soft-textured foods are consumed at a faster rate than firm-textured foods, which require more chewing after each bite [73,74]. Interestingly, when given four different meals consisting of soft minimally processed, soft UPF, hard minimally processed, and hard UPF components, soft textured foods were eaten faster by participants [73]. This accelerated eating rate of soft-textured foods was seen regardless of minimally or ultra-processed status, though soft UPFs were consumed the fastest [73]. Food form is another characteristic that may drive eating rate, as consumption rates of liquid and solid foods differ dramatically [70]. Liquid foods have been shown to have faster consumption rates compared to matched foods in solid forms [75], which may be a way in which UPF beverages (e.g., sodas, energy drinks, pre-packaged shakes) cause increased energy intake [70]. Eating rate remains an important factor in energy consumption as it is more individual to consumers compared to energy density, which is often determined before purchase. Evidence has shown that the modification of eating rates can slow energy intake rates, reducing the risks of overconsumption and weight gain [76,77].

As established, high energy-density and faster eating rates increase the total energy consumption, resulting in a caloric surplus [57,70]. Overeating, particularly with a diet moderately high in added sugar and solid fats, is associated with weight gain and obesity [78,79]. This overconsumption and weight gain may also be further exacerbated by a sedentary lifestyle, which has become increasingly common in the United States [78,80]. Highly palatable foods, which UPFs often are, can offset appetite regulation by up-regulating hunger signals, blunting satiety signals, and activating the reward system, all of which drive consumption for pleasure instead of energy requirements [56]. Eating in response to one’s emotions and mood also increases the risk of overconsumption in some individuals [81]. This is especially true for individuals who are more sensitive to the sensory stimuli of foods, and the resulting overconsumption may be more prevalent during anxiety-inducing times [81]. Further, insulin is elevated by overconsumption, which is thought to lessen the stress-induced responses of adrenocorticotropic hormone (ACTH) and glucocorticoid [82]. Additional mechanisms that are not physiological or psychological in nature have also been explored to explain the phenomenon of UPF overeating [16]. These involve price and availability of the food products, convenience and preparation time to consume, portion size, and how food products are marketed [16]. Overall, the defining properties of high energy density, susceptibility to overeating, and hyperpalatability drive the overconsumption of these food products, further promoting weight gain and increasing risk of chronic disease.

### 2.5. Rise of Ultra-Processed Foods in the United States

The 20th century was a dynamic time for the US food system, characterized by the rise of UPF, which includes the development, industry growth, and heightened consumption from the public. While the advancements to agriculture and food production played a significant role in the increase in UPF, there was also a financial aspect to this growth in the United States. The increased production from the food industry in the US meant prices of some foods dropped [15]. Starting in the late 1940s, this increased production along with the general increase in income among working US Americans, resulted in a slow decrease in the total food expenditures relative to disposable income [15]. At the same time, the total health expenditures relative to disposable income started increasing and surpasses the food spending in the 1980s, reflecting the basic principle of Engel’s Law that amount spent on food decreases as income increases [15]. To combat this and to take advantage of a new, fast-growing industry in the 20th century, there was an effortful push from food corporations to increase their profits through their aggressive marketing tactics, replacement of traditional foods with a variety of new products, and promotion of increased serving sizes and snacking behaviors for more consumption [83]. Another trend in the United States regarding food expenses was the increased spending on foods outside of the home at restaurants, fast food establishments, cafes, and more. Today, the average US adult spends roughly the same amount of food consumed at home as they did in the 1950s, but now they spend around that same amount or more on food eaten outside of the home [84]. Although UPF are not defined by where they are eaten, out-of-home food establishments, especially ones of the fast-food industry, are heavily associated with the prevalence of these foods [85,86].

Recent consumption trend assessments have shown that for the past several decades we have significantly been consuming more UPFs on a global scale [3,4,85,87,88]. In the past twenty years, the US has increased consumption of UPF among adults (53.5 to 57.0% of total kcal), observed between the 2001–2002 and 2017–2018 NHANES survey data sets [4]. Analysis of the 2017–2018 What We Eat in America (WWEIA), NHANES data, NOVA category 4 foods make up 58.2% of the average US adult’s daily caloric intake [5]. Meanwhile, unprocessed/minimally processed foods (Group 1) contributed 27.6%, processed culinary ingredients (Group 2) contributed 5.2%, and processed foods (Group 3) contributed 9.0% of the daily calories [5]. These increased consumption rates on the national and global levels have mirrored another growing trend in the United States, the increased prevalence of obesity. From 1999–2000 to 2017–2018, the prevalence of obesity has risen from 30.5%to 42.4% just in under two decades [89]. Due to these paralleled increases, there has been some focus on the UPFs have on the developments and perpetuation of obesity in the United States [16,90]. The negative health consequences of obesity such as increased risk of developing cardiovascular disease, type 2 diabetes, stroke, and cancer have been well established [91,92,93]. However, it remains unclear if UPFs have a direct role in various negative health outcomes and conditions, or if they facilitate the onset of obesity, which then brings about the health consequences.

## 3. The Relationship Between Ultra-Processed Foods and Disease States

### 3.1. Obesity

#### 3.1.1. Ultra-Processed Foods and Obesity Prevalence

The United States has seen the prevalence of obesity slowly rise over the past several decades, with over 40% of individuals having obesity [94]. This increased prevalence has also been observed on a global scale, yet the US stands out as having the highest level of obesity in children [95]. Numerous health problems may arise from the excess weight, causing obesity, and these consequences include type 2 diabetes, heart disease, stroke, metabolic syndrome, and poor lung health [91]. Ultimately, obesity is now seen as an epidemic that is responsible for an estimated 2.8 million deaths per year worldwide, emphasizing the need for obesity prevention [96]. Consumption of UPFs has seen a similar upward trend over the years, with the rise of popularity starting in the 1960s [3,4]. Ecological data has indicated that the annual per capita UPF consumption has increased ~142% since 1960 [3]. These parallel trends highlight the potential role UPF consumption plays in the development of obesity. Further, lifestyle changes remain a principal recommendation by health care professionals to combat obesity, and this recommendation often has a major emphasis on modifying eating habits [97]. An established relationship between UPFs and obesity may lead to worthy dietary interventions and recommendations for preventing/treating obesity and lowering the obesity rates in the US.

#### 3.1.2. Definition and Etiology of Obesity

Obesity is one of the most common chronic health conditions in the United States and is a major concern in public health due to the increased risk of comorbidities [98]. Obesity is the result of sustained positive energy balance, which occurs when energy intake exceeds expenditure, resulting in weight gain [99]. Traditionally, body weight status has been categorized by body mass index (BMI) measurement. These classifications include BMIs of 25–30 kg/m^2^ as overweight and BMIs of > 30 kg/m^2^ as obesity. Further divisions of obesity based on BMI can be made into subgroups of class 1 (BMI of 30–35 kg/m^2^), class 2 (BMI of 35–40 kg/m^2^), and class 3 (BMI > 40 kg/m^2^) [100]. Obesity is also characterized by the accumulation of lipids (in the form of triglycerides) in adipose tissue, which results in tissue growth and proliferation [101]. Over time, the excess lipids are stored in other areas of the body, mainly in subcutaneous and visceral stores, the latter of which is highly linked with various metabolic disturbances [101]. Alone, BMI is a tool with limitations when it comes to predicting health risks and treatment responses, though waist circumference and body composition metrics used in combination with an elevated BMI for the additional focus on visceral adiposity may be an improved method of categorizing obesity and determining metabolic health risks [98,102].

There is a plethora of causes in the etiology of obesity, involving genetic, environmental, societal, and behavioral factors [98]. Genetically, obesity is highly heritable with some genes being associated with weight gain and increased adiposity [103]. Other less-modifiable conditions such as insomnia, endocrine disorders, Prader–Willi syndrome and MC4R syndromes may also cause obesity [103]. Environmental factors that may drive obesity include food supply changes, availability of palatable foods, decreased activity at home and work, and poverty, which leads to significantly higher rates of obesity [102]. These environmental factors are key factors in the increased prevalence of obesity in children, especially those from impoverished communities [102].

Energy balance is modulated by numerous physiological pathways that influence both energy expenditure and food intake [102]. Resting metabolic rate (RMR) is the required energy for cells and tissue to continue working in a rested state. RMR is a major component of energy expenditure, comprising ~70% of total energy expenditure [104]. This is the heavily determined by skeletal muscle mass, which requires and produces a large amount of energy and contributes to the increased energy expenditure needs [104]. Androgens, regulated by the hypothalamus, can increase skeletal muscle mass, and in mouse models, mice without androgen receptors have reduced energy expenditure rates, which results in late-onset obesity [105]. Meanwhile, physical activity and diet-induced expenditure account for approximately 20% and 10%, respectively, of total energy expenditure [104]. These remaining aspects of energy expenditure rely on mediation via pathways and endocrine cascades involving the brain and peripheral tissue [102]. Neural networks in the stomach and gut detect distention and the presence of nutrients, triggering a hormonal secretion from the organs [102]. These include ghrelin, an appetite-inducing hormone from the stomach, and glucagon-like peptide 1 (GLP-1), peptide YY (PYY), and cholecystokinin (CCK) from the small intestines, all of which induce satiety [102]. The contribution of PYY and CCK to obesity remains unclear as some studies report inconsistent hormone levels pre- and post-meal in individuals with obesity [106]. However, GLP-1 has a more defined role, as individuals with obesity have blunted postprandial GLP-1 levels and these levels normalize after weight loss [106]. Adipose tissue also has a role in hormonal responses to eating, particularly with the release of leptin and adiponectin [102]. These hormones enter circulation to activate agouti-related peptide (AgRP) and proopiomelanocortin (POMC) receptors in the hypothalamus [102]. Leptin modulates satiety through this pathway, and it has been observed in mouse models that mutations resulting in decreased leptin (synthesis, secretion, or activity) causes obesity [107]. Adiponectin plays a role in weight homeostasis, and low concentrations of adiponectin are associated with obesity [108]. This collection of releases and signals is received by the central nervous system, which stimulates other neuronal regions that moderate eating and physical activity [102]. Disturbances in energy balance remain one of the most important determinants of obesity and may be a way by which UPF consumption promotes the onset of obesity.

#### 3.1.3. Ultra-Processed Foods and Obesity—Potential Mechanisms

**Figure 1:**
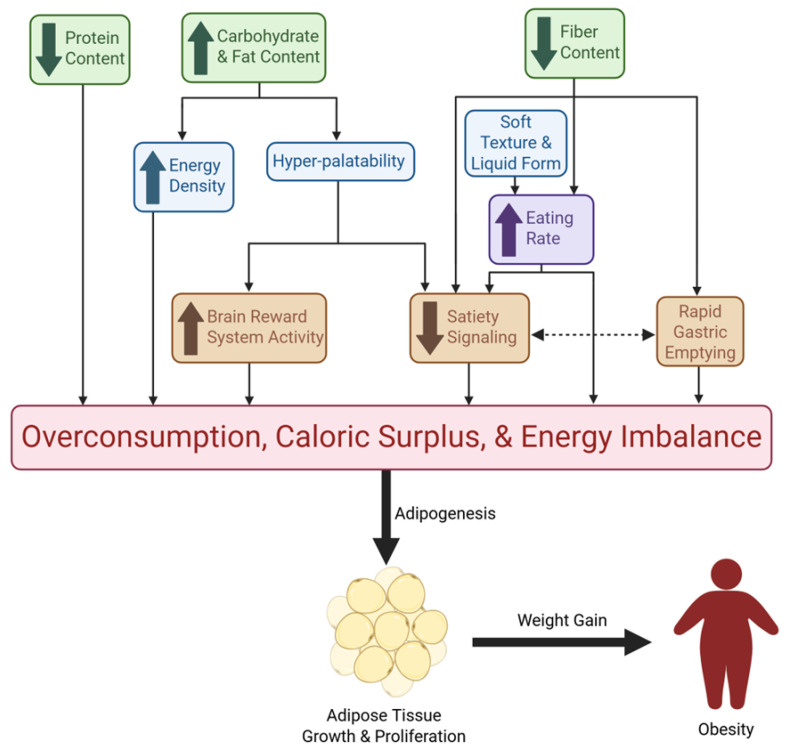
Mechanisms of UPF-Driven Overconsumption and Obesity. This figure illustrates the pathways through which UPFs promote excessive energy intake and obesity. Green boxes represent nutritional characteristics of UPFs; blue boxes denote additional food characteristics commonly seen in UPFs; the purple box reflects a behavioral mechanism; orange boxes represent physiological mechanisms. Collectively, these nutritional, sensory, behavioral, and physiological effects converge on overconsumption, caloric surplus, and energy imbalance. Downstream, chronic excess intake drives adipose tissue growth and proliferation, ultimately contributing to weight gain and obesity. Directional arrows indicate causal and reinforcing relationships among these mechanisms.

A collection of observational studies have found positive associations between greater UPF exposure and greater risk of overweight and obesity [109]. However, a clear mechanism of action as to how UPFs lead to weight gain and obesity is lacking. Despite the numerous factors at play in the development of obesity like genetics, environment, and behavior, UPF consumption may prove to be a significant driving component. UPFs are typically cheaper, more convenient (ready-to-eat), and have longer shelf lives compared to their unprocessed counterparts or foods of origin [6]. These characteristics could be a major driving factor in the choice of food selection and overconsumption, especially for those of lower socioeconomic status, which is commonly associated with higher UPF consumption [110]. Multiple mechanisms have been explored to explain the ways in which UPF-rich diets cause increased caloric intake and offset energy balance, leading to obesity (Figure 1). One plausible mechanism by which UPFs promote obesity is the high energy-dense nature of UPFs which increases ad libitum energy consumption [111]. The most prominent clinical trial investigation on UPFs by Hall and colleagues featured UPF and unprocessed diets that had similar overall energy density due to fiber supplementation in lower-calorie beverages [72]. However, the non-beverage energy density was around 85% higher on the UPF diet compared to the unprocessed diet [72]. This difference in energy density likely played a role in the increased consumption of ~500 kcal/day on the UPF diet, as well as the correlated ~0.9 kg body weight increase [72]. Though this study demonstrated the difference in energy density between non-beverage food items, the UPF diets did not include the typical high-calorie, sugar-sweetened UPF beverages that are commonly consumed [90]. RCT findings show that the inclusion of these beverages in the diet of adults leads to an increase in body weight of ~0.85 kg, and regular consumption likely contributes to the onset of obesity [112].

Increased eating rate is another mechanism that can lead to caloric surplus and is associated with obesity [71]. PYY and GLP-1 are higher 30 min post-meal compared to 5 min post-meal, emphasizing the need for slower eating times for a noticeable loss of appetite before overconsumption [113]. Hall et al. found that the meal eating rate was significantly faster (greater g/min and kcal/min) for the UPF diet than the unprocessed diet [72]. Further, a secondary analysis of this data showed that the eating rate for non-beverage items was a predictor of meal energy consumption for both diets [114]. Eating rate is highly influenced by both food form and texture, with liquids and soft foods being consumed at faster rates [73,75], further driving excess calorie consumption and subsequent weight gain [70,73]. The combination of carbohydrates, fats, salts, and the use of sensory-related industrial additives, such as flavorings, colors, sweeteners, and texturing agents, has led to food characteristics that are not naturally occurring [115,116]. The right combinations of these additives and nutrients create hyperpalatable foods that may override homeostatic feeding mechanisms and promote weight gain via the hedonic pathway [115]. After consumption of highly palatable meals, there is an induced resistance to satiety signals, including CCK, leptin, and insulin, which results in overeating [56]. The attractive taste of hyperpalatable food increases the activity of the brain’s reward system, leading to an altered response by the energy homeostasis mechanisms of the hypothalamus and prolonging intake [56]. In the Hall et al. secondary analysis, it was found that meal energy intake was determined by the portion of calories that came from hyperpalatable foods, which was greater for the UPF diet [114].

An additional way in which high-UPF diets lead to excess caloric intake can be explained by the diluted protein density of UPFs [45]. The low protein density in UPFs results in a greater consumption of calories in order to meet adequate daily protein intake compared to less processed foods that have a greater contribution of protein to total calories [45]. Fiber is another nutrient that is known to be reduced in UPFs [32]. High fiber diets have been shown to reduce the risk of obesity, and this has been explored through various mechanisms [117]. These mechanisms include: 1) increased satiety signals of CCK, GLP-1, and PYY; 2) increased chewing time for full oral digestion; and 3) slowed rates of gastric emptying due to increased viscosity of the digested content, which is also correlated with increased satiation [117,118]. The lack of fiber in many UPFs likely results in an absence of the aforementioned mechanisms, thus explaining overconsumption and obesity. These proposed mechanisms by which UPFs contribute to obesity can be summarized by their contribution to excess energy intake, which promotes lipogenesis and obesity.

### 3.2. Other Chronic Conditions

#### 3.2.1. Obesity, Related Health Concerns, and Ultra-Processed Foods

**Figure 2:**
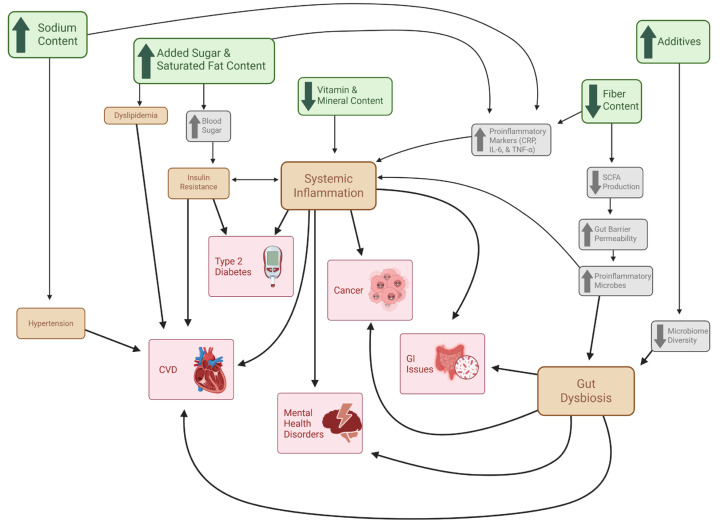
Mechanisms of UPF-Driven Chronic Disease Independent of Obesity. This figure illustrates the proposed pathways through which UPFs contribute to chronic disease development independent of excess adiposity. Green boxes represent characteristic nutritional features of UPFs; gray boxes denote immediate metabolic and physiological responses to these dietary components; orange boxes indicate intermediate pathological states; Red boxes represent downstream chronic health outcomes. Directional arrows depict the interconnected relationships among nutritional exposures, physiological disturbances, intermediate pathologies, and chronic disease development.

Obesity is not only a prevalent disease in the United States, but it also has a significant impact on the risk of developing other noncommunicable chronic diseases [101]. These comorbid diseases include type 2 diabetes, nonalcoholic fatty liver disease, coronary artery disease, heart failure, stroke, and osteoarthritis, among others, and they are heavily driven by the increased adiposity that is characteristic of obesity [101]. Additional mechanisms aside from obesity status have been explored and summarized (Figure 2) to explain the impact of elevated UPF consumption on increased chronic disease risk [119,120,121]. Many of these mechanisms are based on the defining characteristics of UPFs like altered nutrient profile and presence of additives, yet some mechanisms in these investigations are obesity caused by UPF consumption [119]. This makes it unclear if high UPF consumption plays a significant role in chronic disease development alone or if it instead promotes the onset of obesity, which then brings about the well-known comorbidities. In the case of the former, these mechanisms may provide pertinent information to individuals who are not obese yet consume high amounts of UPFs.

#### 3.2.2. Cardiovascular Disease

Cardiovascular disease (CVD) is the leading cause of death in the United States and is a disease that is highly linked with obesity [122]. Many interconnected mechanisms have been described to explain the role obesity has with increased risk of CVD, all stemming from elevated adiposity [101]. One of these mechanisms includes the increased release of free fatty acids (FFAs) from triglyceride (TG) hydrolysis in adipocytes [101]. The elevated FFA levels contribute to dyslipidemia, lipotoxicity, and impaired insulin signaling, all of which directly lead to CVD or contribute to inducive diseases (e.g., type 2 diabetes and coronary artery disease) [101]. UPFs are known to have elevated levels of added sugar [6], and excess sugar intake is highly associated with insulin resistance and dyslipidemia [123]. Another mechanism by which obesity contributes to CVD involves hypertension, which may be caused by mechanical stress of increased adiposity and/or the increased activity of the sympathetic nervous system and the renin–angiotensin–aldosterone system (RAAS) [101]. Increased UPF consumption may also contribute to hypertension due to the increased sodium content of UPFs, a key component driving hyperpalatability [6]. Excessive sodium intake is a risk factor of hypertension and thus increasing CVD risk [119]. High UPF consumption may also increase CVD risk through increased inflammation and gut dysbiosis [119]. These mechanisms and the role UPFs have in them will be explored in subsequent sections in order to explore additional associated chronic diseases. The role that UPF consumption has in CVD risk is influential and should be another reason for the overall reduction in UPF consumption in order to avoid chronic disease and promote health.

#### 3.2.3. Inflammation

Not only do foods influence the homeostatic and hedonic pathways through their addictive-like properties, but they may also contribute to brain inflammation [72,124,125]. According to Lin and Qu (2020), obesity commonly co-occurs in the setting of inflammation, with inflammation occurring in the CNS before peripheral tissues [124]. High-fat diet exposure has been shown to activate specific cytokines and inflammatory pathways in the hypothalamus, leading to neuronal injury and reducing hypothalamic insulin sensitivity [124]. Dietary fat, in particular long-chain saturated fatty acids (SFAs), can cross the BBB and accumulate in the hypothalamus [125]. It has been found that SFAs can activate specific proinflammatory signaling cascades, leading to inflammation, and blunting leptin and insulin signaling within the hypothalamus [125]. The BBB, an intermediate between the brain and the periphery, is also negatively influenced by high-fat diets [125]. It has been reported that overnutrition and peripheral inflammation can decrease the expression of tight junctions and increase vascular endothelial growth factor within the BBB, leading to increased permeability and promoting BBB integrity disruption [125]. The hippocampus is another brain structure that is impacted by inflammation despite being fully protected by the BBB [lin]. Prolonged inflammation can lead to long-lasting impaired metabolic control of the hypothalamus [lin]. If the primary center of the brain that regulates appetite is compromised, then regulating food and calorie intake will be negatively impacted, leading to obesity [124,125].

Chronic inflammation is a state of the body that can lead to metabolic dysfunctions, leading to favorable conditions for the onset of diseases like CVD, diabetes, and cancer [120]. Further, it has been demonstrated that depression and fatigue are closely linked to increased peripheral and central immune activation [126]. Increased adiposity in obesity leads to increased proinflammatory adipokine synthesis which increases tissue macrophages and leads to low-grade systemic inflammation [101]. This inflammatory state may then lead to increased insulin resistance, causing type 2 diabetes, nonalcoholic fatty liver disease, cirrhosis, and CVD [101]. UPFs may promote an inflammatory state through various mechanisms, particularly through their influence on C-reactive protein (CRP), an inflammatory biomarker widely studied with UPF consumption [120]. Surprisingly, the Hall et al. clinical trial showed that the UPF-rich diet did not significantly change CRP levels, but the unprocessed diet significantly reduced them [72]. UPFs typically have high amounts of simple sugars (e.g., sucrose and high-fructose corn syrup) that raise blood sugar and increase insulin levels, resulting in a proinflammatory state [120]. Further, sugar-sweetened beverages have been associated with increased levels of CRP and IL-6 [127]. High CRP levels in adults have also been associated with elevated salt intake [128]. The increased fat content of UPFs and their poor-quality fats, specifically trans fatty acids, are associated with elevated CRP, IL-6, and TNF-α levels [120]. UPFs are also characterized by their decreased dietary fiber and micronutrient content [31]. Fiber is nutrient that has been shown to maintain low CRP levels by maintaining gut microbiome homeostasis [129]. Several micronutrients such as vitamin C, vitamin D, zinc and niacin have anti-inflammatory factors [120]. These nutritional aspects of UPFs highlight the impactful role increased UPF consumption has, independent of obesity, on systematic inflammation and associated chronic diseases.

#### 3.2.4. Gut Dysbiosis

The gut microbiome is a complex feature of the digestive system that plays fundamental roles in various body processes, including metabolism, immunity, and cognition [121]. Disruptions in the gut microbiome have been associated with chronic health conditions like obesity, type 2 diabetes, CVD, and mental health disorders [121]. The lack of fiber in UPFs is pernicious to the gut microbiome, as fiber is fermented by gut bacteria to generate short-chain fatty acids (SCFA) to maintain gut health [130]. SCFAs provide energy for colonocytes and maintain intestinal barrier integrity through mucin secretion, tight junctions, low pH, and antimicrobial production [121]. UPFs may lead to reduced SCFA production due to low fiber content, which weakens the intestinal barrier and increases gut permeability, allowing proinflammatory microbes to enter circulation [121]. Various additives (e.g., preservatives, emulsifiers, artificial flavorings) found in UPFs are associated with microbiome disturbance, specifically reduction in microbial α-diversity, an important part of gut microbiome balance [39]. Irritable bowel disease and colorectal cancer are both associated with reduced microbiome and increased proinflammatory microbes [121]. The gut–brain axis describes the close connection between cognitive health and the gut microbiota, and how the interaction between the organ systems promotes homeostasis [131]. Disruptions in the gut microbiome can lead to neurologically associated disorders like depression, Alzheimer’s disease, and Parkinson’s disease [121]. It is possible that increased UPF consumption also influences this aspect of gut health through the aforementioned mechanisms. An umbrella review investigating UPF-associated health risks found that greater UPF consumption was highly associated with depression, anxiety, and mental disorder outcomes [109]. UPF consumption has a significant impact on gut health due to the dietary qualities of the foods, and the resulting environment is favorable for dysbiosis and inflammation, increasing the risk of chronic disease development.

## 4. Future Directions

Previous investigations have shown that increased UPF intake is associated with various diseases and health concerns, including obesity, CVD, type 2 diabetes, mental health disorders, and more [103]. The poor nutritional quality of UPFs likely promotes overconsumption through various mechanisms, which leads to obesity and related cardiometabolic diseases (Figure 1). There is a lack of studies focused on the benefits of dietary intervention based on the reduction in UPFs. The well-known Hall et al. clinical trial that investigated the effects of an unprocessed diet versus a diet rich in UPFs has been the only RCT conducted on this topic [66]. While the results of this study demonstrated relevant findings on UPF-rich diets and their role in caloric overconsumption and weight gain, the study was highly controlled with in-patient housing and all meals provided [66]. Therefore, there is still a need for the investigation for the effects of UPF reduction in free-living conditions. The feasibility of this dietary intervention remains unknown and may be a more difficult task considering the abundance of UPFs found in the typical Western diet [4] as well as the complexity of understanding UPF categorization. An investigation is required to determine whether or not successful UPF reduction in free-living conditions leads to positive health outcomes and decreased chronic disease risk. Further research should also explore practical, scalable intervention strategies that could support individuals in reducing UPF intake. Potential approaches may include behavioral nutrition interventions (e.g., goal setting, self-monitoring) to help individuals replace UPFs with minimally processed alternatives; supermarket-based interventions (e.g., front-of-package labeling) to help consumers identify UPFs; culinary skill building to improve cooking confidence and decrease reliance on UPFs; or digital/app-based tools to quickly and conveniently classify foods based on processing level.

## 5. Conclusions

The evidence reviewed here demonstrates that ultra-processed foods influence metabolic health through interconnected behavioral, hormonal, microbial, and biochemical mechanisms that extend well beyond nutrient composition alone. UPFs promote rapid eating, high energy intake, and nutrient displacement, while simultaneously impairing key metabolic regulators such as appetite hormones, inflammatory pathways, and gut-derived metabolites. These effects converge on metabolic processes central to cardiometabolic disease, including insulin sensitivity, adipose tissue function, and chronic low-grade inflammation. Although observational data consistently link UPF consumption to obesity, cardiovascular disease, and type 2 diabetes, mechanistic understanding remains incomplete. The emerging evidence presented in this review highlights plausible biological pathways, yet targeted human intervention studies are urgently needed to clarify the causal impact of reducing UPF intake on metabolic biomarkers and long-term risk. Integrating metabolomics, microbiome analyses, and metabolic pathway mapping into future designs will be essential to advancing this work. Overall, the available data support the view that food processing level is a meaningful determinant of metabolic health. Recognizing the mechanistic impact of UPFs offers an important opportunity for refining dietary guidelines, informing public health strategies, and developing interventions aimed at improving cardiometabolic outcomes in diverse populations.

## Data Availability

No new data were created or analyzed in this study. Data sharing is not applicable to this article.

## Abbreviations

The following abbreviations are used in this manuscript:

UPF	Ultra-processed food
BMI	Body mass index
CVD	Cardiovascular disease
ACTH	Adrenocorticotropic hormone
GLP-1	Glucagon-like peptide-1
PYY	Peptide YY
CCK	Cholecystokinin
AgRP	Agouti-related protein
POMC	Proopiomelanocortin
FFA	Free fatty acid
TG	Triglyceride
SCFA	Short-chain fatty acid
RMR	Resting metabolic rate
IL-6	Interleukin-6
TNF-α	Tumor necrosis factor-α
CRP	C-reactive protein
RAAS	Renin–angiotensin–aldosterone system
CNS	Central nervous system
